# The Interaction Between Nodal, Hypoxia-Inducible Factor 1 Alpha, and Thrombospondin 1 Promotes Luteolysis in Equine Corpus Luteum

**DOI:** 10.3389/fendo.2019.00667

**Published:** 2019-10-01

**Authors:** Edyta Walewska, Karolina Wołodko, Dariusz Skarzynski, Graça Ferreira-Dias, António Galvão

**Affiliations:** ^1^Department of Reproductive Immunology and Pathology, Institute of Animal Reproduction and Food Research, Polish Academy of Sciences, Olsztyn, Poland; ^2^The Centre for Interdisciplinary Research in Animal Health, Faculty of Veterinary Medicine, University of Lisbon, Lisbon, Portugal

**Keywords:** Nodal, hypoxia inducible factor 1 alpha, thrombospondin 1, prostaglandin F2 alpha, corpus luteum, luteolysis

## Abstract

The regulation of corpus luteus (CL) luteolysis is a complex process involving a myriad of factors. Previously, we have shown the involvement of Nodal in functional luteolysis in mares. Presently, we ask the extent of which Nodal mediation of luteolysis is done through regulation of angioregression. We demonstrated the interaction between Nodal and hypoxia-inducible factor 1 α (HIF1α) and thrombospondin 1/thrombospondin receptor (TSP1/CD36) systems, could mediate angioregression during luteolysis. First, we demonstrated the inhibitory effect of Nodal on the vascular marker platelet/endothelial cell adhesion molecule 1 (CD31). Also, treatment of mid CL explants with vascular endothelial growth factor A (VEGFA) showed a trend on activin-like kinase 7 (Alk7) protein inhibition. Next, Nodal was also shown to activate HIF1α and *in vitro* culture of mid CL explants under decreased oxygen level promoted Nodal expression and SMAD family member 3 (Smad3) phosphorylation. In another experiment, the crosstalk between Nodal and TSP1/CD36 was investigated. Indeed, Nodal increased the expression of the anti-angiogenic TSP1 and its receptor CD36 in mid CL explants. Finally, the supportive effect of prostaglandin F2α (PGF2α) on TSP1/CD36 was blocked by SB431542 (SB), a pharmacological inhibitor of Nodal signaling. Thus, we evidenced for the first time the *in vitro* interaction between Nodal and both HIF1α and TSP1 systems, two conserved pathways previously shown to be involved in vascular regression during luteolysis. Considering the given increased expression of Nodal in mid CL and its role on functional luteolysis, the current results suggest the additional involvement of Nodal in angioregression during luteolysis in the mare, particularly in the activation of HIF1α and TSP1/CD36.

## Introduction

Molecular regulation of luteolysis is a very intricate process (1). Following the trigger of the uterine prostaglandin (PG) F_2α_, various local auto-, paracrine interactions are initiated (1, 2). Amongst others, the morphogens from transforming growth factor-β (TGF β) superfamily Nodal and TGFβ1 appear to be key for luteolysis in the mare (3). Particularly Nodal, after binding to its type II receptor, the activin-A receptor 2B (ACVR2B), phosphorylates either activin-like kinase 4 (Alk4) or activin-like kinase 7 (Alk7), and subsequently mediates the phosphorylation of a SMAD family member 2 (Smad2) or Smad3, which finally translocates to the nucleus and regulates transcription (4). We have recently shown that Nodal enters a close feed-forward loop with PGF_2α_ toward progesterone (P4) downregulation and intraluteal PGF_2α_ amplification at the time of luteolysis initiation (2). Importantly, when we blocked Nodal and TGFβ1 signaling in PGF_2α_ treated cells we abolished its functional and structural luteolytic role (3). Furthermore, the regression of the corpus luteum (CL) is also associated with decreased blood flow (5, 6), which originates low oxygen (O_2_) tension in the organ, an event named hypoxia (7). In response to hypoxic conditions, cells develop different strategies such as the transcription of hypoxia-inducible factor 1 (HIF1). Indeed, HIF1 consists of two subunits: (i) HIF1β, which is constitutively expressed in the nucleus; and (ii) HIF1α, which responds to different factors like cellular O_2_ tension or other cytokines (7). Importantly, HIF1α has been linked to both functional and structural luteolysis (8, 9).

Additionally, a well-characterized anti-angiogenic factor is the thrombospondin 1 (TSP1) (10). Previous studies revealed the reciprocal inhibitory action between TSP1 and the proangiogenic fibroblast growth factor 2 (FGF) (11). Thrombospondin 1 belongs to a family of five conserved glycoproteins that are associated with cell-to-cell and cell-matrix interactions (11, 12). The ligand TSP1 and its receptor cluster of differentiation 36 (CD36) were shown to be widely expressed in the ovary, mainly in granulosa cells, large steroidogenic cells, and endothelial cells (13). Indeed, TSP1/CD36 system was shown to promote luteal endothelial cells apoptosis and in this way inhibit angiogenesis (14).

The present study investigates the putative involvement of Nodal in vascular regression in the mare. Taking advantage of an *in vitro* model with mid CL explants, presenting cell-to-cell interactions that are absent in luteal cell systems, we studied the crosstalk between Nodal signaling various vasoactive mediators. Thus, it was assessed: (i) the effect of Nodal on the marker cluster of differentiation 31 (CD31) protein, and, conversely, Nodal signaling protein components regulation by vascular endothelial factor A (VEGFA) and FGF; (ii) HIF1α profile in early, mid, and late CL and the effect of Nodal treatment on HIF1α expression in mid CL explants; (iii) the extent of which hypoxia activates Nodal signaling in mid CL explants; (iv) if the putative crosstalk between Nodal and HIF1α includes VEGFA activity; and (v) Nodal regulation of TSP1/CD36 system, as well as Nodal supportive role on PGF_2α_-mediated amplification of TSP1 and CD36 proteins.

## Materials and Methods

### Equine Corpus Luteum Collection

All procedures for animal handling and tissue collection were approved by the Local Animal Care and Use Committee in Olsztyn, Poland (Agreement No. 51/2011). The mares used in this study (aged 3–8 years) were declared clinically healthy by the official government veterinary inspector and by individual historical records of animal health. After stunning, mares were euthanized, according to European Legislation concerning welfare aspects of animal stunning and euthanasia methods (EFSA, AHAW/04-027). Genitalia were collected *post-mortem* at the abattoir. As previously described, mare luteal samples were collected from April until the end of July and classified based on the morphological appearance of the CL, the presence of follicles in the ovary and plasma P4 concentration as: early luteal phase CL (early CL; presence of corpus hemorrhagicum, P4 < 1 ng/mL), mid luteal phase CL (mid CL; CL associated with follicles 15–20 mm in diameter, P4 > 6 ng/mL), and late luteal phase CL (late CL; CL associated with preovulatory follicle 30–35 mm in diameter, P4 between 1 and 2 ng/mL) (15). Immediately after collection, luteal samples were placed in specific solutions: (i) RNAlater (AM7020; Ambion, Carlsbad, USA) for gene (*n* = 6) and protein (*n* = 6) expression quantification; (ii) transport media M199 (M2154; Sigma-Aldrich, Saint Louis, USA) with 20 μg/mL gentamicin (G1397; Sigma-Aldrich) for *in vitro* studies.

### An *in vitro* Culture for Mid CL Explants

Corpora lutea from mid luteal phase (*n* = 6) were washed in phosphate buffered saline (PBS) 0.1 M (pH = 7.4) supplemented with 20 μg/mL gentamicin and minced into small pieces of ~1 mm^3^ and 30 mg weight. Luteal explants (30 mg) were then cultured in 1 mL of Dulbecco's modified eagle's medium (DMEM) and F-12 Ham medium (D/F medium; D-8900; Sigma-Aldrich) containing 10% fetal bovine serum (FBS) (26140-079, ThermoFisher-Scientific, Waltham, USA), 20 μg/mL gentamicin and 250 μg/mL amphotericin (A2942, Sigma-Aldrich), in 24 well-culture plates, at 37°C in humidified atmosphere (5% CO_2_, 95% air). After stabilization for 1 hour (h), culture media was changed with fresh one and mid CL explants cultured for 24 h and treated differently.

To assess the effect of Nodal treatment on proangiogenic factor (CD31), mid CL explants were treated as (i) no factor (negative control); (ii) Nodal (10 ng/mL, 3218-ND-025, R&D Systems, Minneapolis, USA); (iii) PGF_2α_ (10^−7^ M, P0424-1MG, Sigma Aldrich); and (iv) luteinizing hormone (LH) (10 ng/mL, L9773; Sigma). Next, in order to examine Nodal signaling responsiveness to proangiogenic factors, mid CL explants were exposed to (i) no factor (negative control); (ii) VEGFA (selected dose 25 ng/mL—doses tested 1, 10, and 25 ng/mL, V7259, Sigma); (iii) FGF (selected dose 10 ng/mL—doses tested 1, 10, and 25 ng/mL, SRP3040, Sigma); and (iv) LH (10 ng/mL). Subsequently, in order to study the crosstalk between Nodal and HIF1α, mid CL explants were treated with (i) no factor (negative control); (ii) Nodal (0.1, 1, and 10 ng/mL); (iii) PGF_2α_ (10^−7^ M); and (iv) LH (10 ng/mL). Additionally, we assessed VEGFA mRNA and protein levels. In another experiment, the crosstalk between Nodal and TSP1 system was studied, and TSP1 and CD36 expression analyzed after treating mid CL explants with (i) no factor (negative control); (ii) Nodal (10 ng/mL); (iii) PGF_2α_ (10^−7^ M); and (iv) LH (10 ng/mL). Finally, we confirmed the requirement of Nodal signaling during the PGF_2α_ upregulation of TSP1/CD36, as mid CL explants were treated with (i) no factor (negative control); (ii) SB431542 (SB) (10 μM, 1614/1, R&D Systems); (iii) PGF_2α_ (10^−7^ M); and (iv) simultaneously SB (10 μM) and PGF_2α_ (10^−7^ M). Both PGF_2α_ and LH treatments of mid CL explants represented internal controls. Mid CL explants after treatment were stored in RNAlater or Radioimmunoprecipitation Assay buffer (RIPA) buffer (89901, Life-Technologies, Carlsbad, USA) at −80°C until mRNA and protein expression analysis was performed.

### An *in vitro* Culture for Mid CL Explants Under Hypoxia

In order to characterize Nodal signaling activation under hypoxic conditions, mid CL explants were cultured in normoxia (20% O_2_) or hypoxia (5% O_2_). After 12 h of tissue culture in normoxia, the medium was replaced and cultures were subjected for 24 h to either: (i) 20% O_2_; (ii) or 5% O_2_ at 37.5°C in a N_2_-O_2_-CO_2_-regulated incubator (ESPEC Corp., Osaka, Japan; no. BNP- 110) as described before (8). Subsequently, mid CL explants were stored in RNAlater or RIPA at −80°C for mRNA and protein expression analysis.

### The Assessment of Mid CL Explants Viability

Tissue viability was assessed with Alamar-Blue Assay (Alamar-Blue, Serotec, UK) (*n* = 4–6) (16). After *in vitro* culture, the Alamar-Blue reagent was added to 24 well-plates and incubated for 4 h in 37°C. Plates were read at 560 nm wavelength. Cell viability in control wells (without any reagent) was considered 100%.

### Real-Time PCR

Total RNA was extracted from either fresh CL tissues (early CL, *n* = 6; mid CL, *n* = 6; late CL, *n* = 6) or after *in vitro* culture of mid CL explants (mid CL, *n* = 6) using Trizol (T9424, Sigma-Aldrich) (16). Briefly, the tissue was minced with homogenizer in Trizol, incubated for 5 min in room temperature (RT) followed by centrifugation at 9,400 g, 4°C for 15 min and collection of supernatant to the new tube. Then, solution was thoroughly mixed with 1-Bromo-3-chloropropan (BCP, BP151, Molecular Research Centre, Cincinnati, Ohio, USA), incubated in RT for 10 min and centrifuged (13,500 g, 15 min, 4°C). The aqueous phase was mixed with isopropanol (190764, Sigma Aldrich), incubated in −80°C for 60 min, centrifuged (20,000 g, 15 min, 4°C) followed by multiple washes with 75% ethanol. On the next day samples concentrations and purity were measured on NanoDrop and the ratio between absorbance at 230, 260, and 280 nm was calculated and confirmed good quality and quantity of extracted RNA. Reverse transcription was performed with 1.5 μg RNA according to the manufacturer's instructions (A15299; Applied-Biosystems, Warrington, UK). The cDNA was stored in −20°C until real-time Polymerase Chain Reaction (PCR) was performed. Then real-time PCR was performed in a 7900 Real-Time PCR System (Applied Biosystems) (primers in Table 1) as described before (16), using Maxima SYBR Green/ROX qPCR Master Mix (K0223, ThermoScientific). The primers were designed using Primer 3.0 v.0.4.0. software (17, 18), based on gene sequences in GeneBank (NCBI). All primers were synthesized by Sigma Aldrich and validated before running experimental samples. Two different primers concentration were tested (80 or 160 nM). The melting curves after each run were obtained by stepwise increases from 60 to 95°C, in order to ensure a single product amplification. Primer concentration was chosen based on the lowest cycle threshold value and the highest melting temperature (Tm) for the product. Primers were also tested for dimers formation. Target gene and a reference gene β*2 microglobulin (B2MG)* were run simultaneously. The total reaction volume was 12 μL, containing 4 μL cDNA (10 μg), 1 μL each forward and reverse primers (80 or 160 nM), and 6 μL SYBR Green PCR master mix. Real-time PCR was carried out as follows: initial denaturation (10 min at 95°C), followed by 45 cycles of denaturation (15 s at 95°C) and annealing (1 min at 60°C). After each PCR reaction, melting curves were obtained by stepwise increases in temperature from 60 to 95°C to ensure single product amplification. Real-time PCR results were analyzed with the Real-time PCR Miner algorithm (19).

**Table 1:** Specific primers sequences used for quantitative real-time PCR.

**Gene name**	**Gene symbol**	**GeneBank accession no**.	**Sequences 5^**′**^–3^**′**^**	**Length (base pair)**
Cluster of differentiation 36	*CD36*	XM_001487907.1	For: AACCACACCGTCTCCTTCGT	107
			Rev: GCCGCTACAGCCAGATTGAG	
Hypoxia inducible factor 1α	*HIF1α*	XM_023627857.1	For: CCAAAAGCCGAAATCCCTTC	80
			Rev: CCAGCCCACGTCTTCTCCTA	
Thrombospondin 1	*TSP1*	XM_001503599.2	For: GCTCCAGCTCTACCAATGTCCT	91
			Rev: TTGTGGCCGATGTAGTTAGTGC	
Vascular endothelial growth factor A	*VEGFA*	NM_001081821	For: ATGCGGATCAAACCTCACCA	117
			Rev: AGGCCCACAGGGATTTTCTT	
β-2 microglobulin	*B2mg*	X69083	For: CGGGCTACTCTCCCTGACTG	92
			Rev: AACCGAAAGGTAAGAGACGAC	
			Rev: GGGACGAGGTTGTCCTGGTA	

### Western Blot

Fresh CL tissue (early CL, *n* = 6; mid CL, *n* = 6; late CL, *n* = 6) and *in vitro* tissue explants (mid CL, *n* = 6) were disrupted by homogenization in RIPA (250 μL) containing protease inhibitor (P8340, Sigma-Aldrich), phospho-stop solution (88667, ThermoFisher) and phenylmethylsulfonyl fluoride (PMSF) (P7626, Sigma-Aldrich) at 4°C. Protein concentration was determined with bicinchoninic acid assay (BCA) (BCA1-1KT, Sigma-Aldrich). A total of 10–80 μg of protein was run on 6–12% (varying accordingly to each protein) polyacrylamide gel followed by transfer to nitrocellulose membranes. Then, membranes were incubated with primary antibodies (Table 2) at 4°C, overnight. Goat anti-mouse alkaline phosphatase conjugated antibodies (1:30,000, 31321, ThermoFisher), goat anti-rabbit alkaline phosphatase-conjugated antibodies (1:30,000, A3687, Sigma-Aldrich), and rabbit anti-goat alkaline phosphatase-conjugated antibodies (1:30,000, A4187, Sigma-Aldrich) were used as a secondary antibody. Immune complexes were visualized using alkaline phosphatase substrate. Blots were scanned in Molecular Imager VersaDoc MP 4000 System (BioRad, Hercules, California, USA) and specific bands quantified using ImageLab Software (BioRad). At last, band density for each of the target protein was normalized against β-actin.

**Table 2:** Specification of antibodies used for western blot.

**Antibody name and specificity**	**Cat no, company**	**RRID no**.	**Antibody dilution**
Mouse monoclonal against HIF1α	ab16066, Abcam, UK	RRID:AB_302234	1:200
Mouse monoclonal against Nodal	ab55676, Abcam	RRID:AB_2151660	1:400
Mouse monoclonal against TSP1	ab1823, Abcam	RRID:AB_2201948	1:100
Rabbit polyclonal against Smad3	ab73942, Abcam	RRID:AB_1566742	1:500
Rabbit polyclonal against Smad3 Phosphorylated	ab51451, Abcam	RRID:AB_882595	1:1000
Rabbit polyclonal against CD31	ab28364, Abcam	RRID:AB_726362	1:200
Rabbit polyclonal against Alk7	ab71539, Abcam	RRID:AB_1267623	1:100
Rabbit polyclonal against VEGFAA	sc-152, Santa Cruz Biotechnology, Dallas, USA	RRID:AB_2212984	1:200
Goat polyclonal against CD36	sc-5522, Santa Cruz Bioetchnology	RRID:AB_638143	1:200
Mouse monoclonal against β-actin	A2228, Sigma Aldrich	RRID:AB_476697	1:10000

### Plasma Progesterone Analysis

Progesterone levels were determined as before (15). The antiserum was used at the final dilution of 1:100 000, and HRP-labeled P4 was used at the concentration of 1:75 000. The standard curve ranged from 0.39 to 100 ng/mL, and the concentration of P4 at 50% binding (ED50) was 4.3 ng/mL. Finally, intra- and interassay coefficients of variation were 5.8 and 8.5%, respectively.

### Statistical Analysis

The data are shown as mean ± SEM of values obtained in separate experiments, each performed in triplicate. Statistical analysis was performed using GraphPad Prism 7.0. In all experiments, samples were tested for normality with the D'Agostino-Pearson omnibus normality test. The real-time PCR and western blot results obtained from studies on fresh CL tissue were analyzed using non-parametric one-way ANOVA Kruskal-Wallis followed by Dunn's multiple comparison test. The analysis of mRNA and protein expression from *in vitro* experiments was performed using non-parametric Friedman test with Dunn's multiple comparison test. The tissue viability was analyzed using Wilcoxon test (Graph Pad Software version7, San Diego, USA). Significance was defined as *p* < 0.05.

## Results

### Nodal Downregulates the Vascular Marker CD31

**Figure 1:**
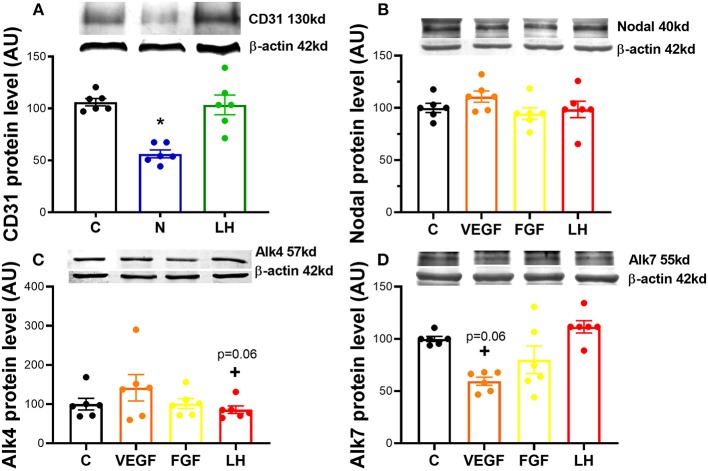
Nodal interaction with proangiogenic factors. **(A)** Expression of proangiogenic cluster of differentiation 31 (CD31) protein after 24 h culture: (i) no factor (C, negative control); (ii) Nodal (10 ng/mL); and (iii) luteinizing hormone (LH, 10 ng/mL). Expression of angiogenic factors on **(B)** Nodal, **(C)** activin receptor (Alk) type 1B (Alk4) and **(D)** type 1C (Alk7) after 24 h culture with: (i) C (negative control); (ii) vascular endothelial growth factor A (VEGFA, 25 ng/mL); (iii) fibroblast growth factor (FGF, 10 ng/mL); (iv) luteinizing hormone (LH, 10 ng/mL). Protein expression determined by western blot, upper panel: representative immunoblot; lower panel: densitometry of protein expression relative to β-actin expression. (*n* = 6). Values are expressed as means ± SEM in arbitrary units (AU). Statistical differences marked with an asterisk (*p < 0.05).

We first asked if Nodal was able to modulate angiogenic factors expression, and confirmed the level of CD31 protein was significantly downregulated in *in vitro* culture of mid CL explants treated with Nodal (*n* = 6) (Figure 1A; *p* < 0.05). Conversely, when we treated mid CL explants with VEGFA, we found no changes in Nodal (Figure 1B) and Alk4 (Figure 1C), but a trend was visible on Alk7 expression inhibition (Figure 1D; *p* = 0.06). Finally, FGF did not affect Nodal or the receptors Alk4, Alk7 (Figures 1B–D).

### Nodal Stimulates HIF1α and Responds to Oxygen Levels

**Figure 2:**
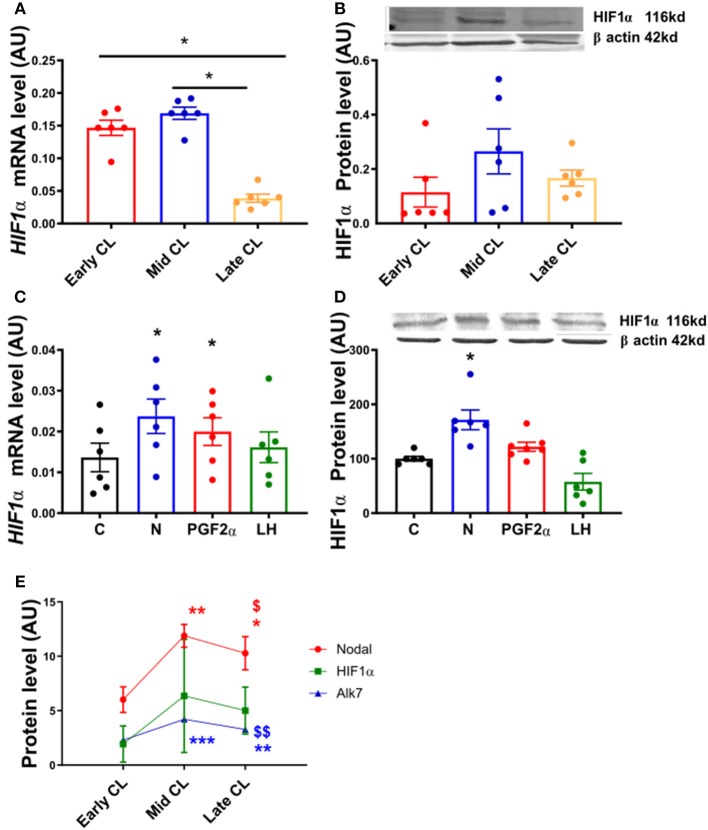
HIF1α mRNA and protein expression in fresh CL explants and after *in vitro* culture of mid CL explants. **(A)** *Hypoxia inducible factor 1* α *(HIF1*α*)* mRNA and **(B)** HIF1α protein expression in early, mid and late CL explants (*n* = 6). Expression of **(C)** *HIF1*α mRNA and **(D)** HIF1α protein after 24 h culture: (i) no factor (C, negative control); (ii) Nodal (10 ng/mL); (iii) prostaglandin F2α (PGF_2α_, 10^−7^ M); and (iv) luteinizing hormone (LH, 10 ng/mL) (*n* = 6). **(E)** Nodal, HIF1α, and Alk7 protein level in early, mid and late CL explants. mRNA expression determined by real-time PCR, expression relative to β*-2-microglobulin (B2MG)* expression. Protein expression determined by western blot, upper panel: representative immunoblot; lower panel: densitometry of protein expression relative to β-actin expression. Values are expressed as means ± SEM in arbitrary units (AU). Statistical differences are marked with asterisks (*p < 0.05). **(E)** *Significant differences with regard to early CL; ^$^significant differences with regard to mid CL (one symbol, *p* < 0.05; two symbols *p* < 0.01).

**Figure 3:**
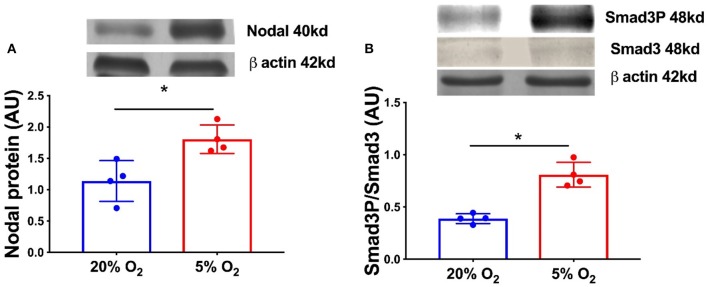
Nodal signaling in mid CL explants cultured *in vitro* under hypoxia. Effect of hypoxia on **(A)** Nodal protein expression and **(B)** phosphorylation of Smad3 in mid CL explants cultured under 20% O_2_ or 5% O_2_ for 24 h (*n* = 4). Protein expression determined by western blot, upper panel: representative immunoblot; lower panel: densitometry of protein expression relative to β-actin expression. Values are expressed as means ± SEM in arbitrary units (AU). Statistical differences are marked with asterisks (*p < 0.05).

**Figure 4:**
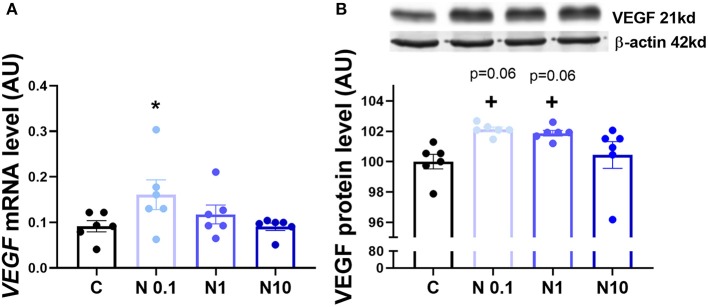
VEGFA mRNA and protein expression after *in vitro* culture of mid CL explants. Expression of **(A)** *vascular endothelial growth factor A (VEGFA)* mRNA, **(B)** VEGFA protein after 24 h culture: (i) no factor (C, negative control); (ii) Nodal (0.1 ng/mL); (iii) Nodal (1 ng/mL); (iv) Nodal (10 ng/mL) (*n* = 6). mRNA expression determined by real-time PCR, expression relative to β*-2-microglobulin (B2MG)* expression. Protein expression determined by western blot, upper panel: representative immunoblot; lower panel: densitometry of protein expression relative to β-actin expression. Values are expressed as means ± SEM in arbitrary units (AU). Statistical differences are marked with asterisks (*p < 0.05).

In order to confirm the expression of HIF1α in equine CL, we performed real-time PCR and western blot in fresh CL samples from early, mid and late CL samples (*n* = 6 for each stage of luteal phase) isolated from cyclic animals. We found that transcription of *HIF1*α peaked in early and mid CL and decreased in late CL, whereas protein did not change significantly (Figures 2A,B; *p* < 0.05). Next, we investigated the direct interaction between Nodal and HIF1α *in vitro* and verified that Nodal (10 ng/mL) stimulated *HIF1*α mRNA and protein expression (Figures 2C,D; *p* < 0.05). Moreover, we plotted the protein levels of Nodal and its receptor Alk7 against HIF1α and found a similar signature throughout luteal phase, with a sharp raise from early CL to mid CL, and a slight drop in late CL (Figure 2E; *p* < 0.01, *p* < 0.05, respectively). We than tested these results manipulating the availability of O_2_, and exposed the mid CL explants to different O_2_ levels. We discovered that Nodal was actively upregulated and Smad3 was phosphorylated when the O_2_ level decreased from 20 to 5% (Figures 3A,B; *p* < 0.05). Neither Nodal treatment nor O_2_ level affected the viability of CL tissues (Supplementary Figures 1A–C). In the last part of the experiment we questioned if the stimulatory effect of Nodal on HIF1α affected its main target, the VEGFA. We found that lower doses of Nodal (0.1 ng/mL) were able to amplify *VEGFA* mRNA (Figure 4A; *p* < 0.05) and VEGFA protein was slightly increased (*p* = 0.06) for the treatments 0.1 and 1 ng/mL, but no effect after Nodal 10 ng/mL treatment (Figure 4B).

### Nodal Crosstalk With TSP1 and CD36 in Mid CL Explants

**Figure 5:**
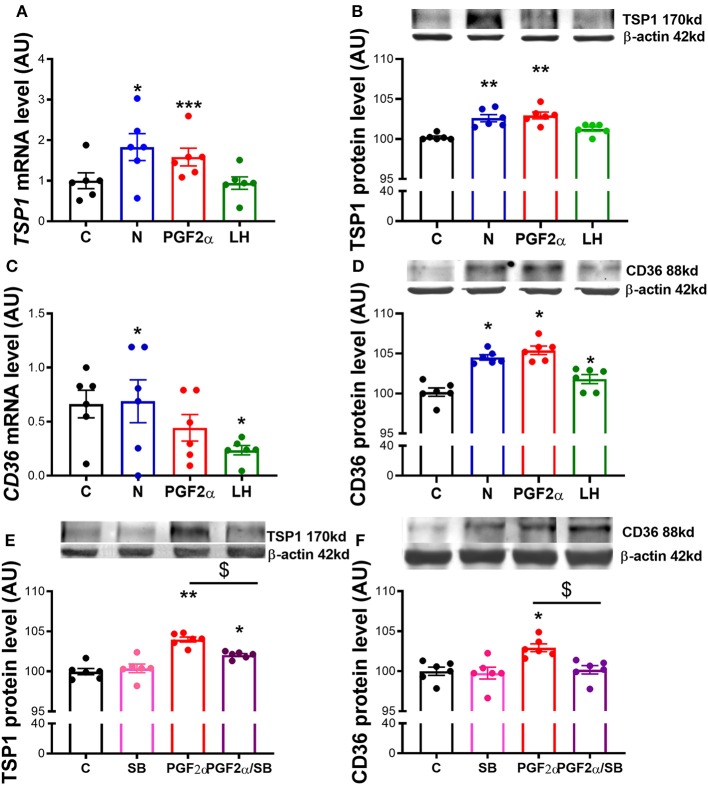
Nodal crosstalk with TSP1 and CD36 in mid CL explants cultured *in vitro*. Expression of **(A)** *thrombospondin 1 (TSP1)* mRNA, **(B)** TSP1 protein, **(C)** *cluster of differentiation 36 (CD36)* mRNA and **(D)** CD36 protein after 24 h culture: (i) no factor (C, negative control); (ii) Nodal (10 ng/mL); (iii) prostaglandin F2α (PGF_2α_ 10^−7^ M); and (iv) luteinizing hormone (LH, 10 ng/mL) (*n* = 6). Expression of **(E)** TSP1 protein and **(F)** CD36 protein after 24 h culture: (i) no factor (C, negative control); (ii) SB (10 μM); (iii) PGF_2α_ (10^−7^ M); and (iv) simultaneously SB (10 μM) and PGF_2α_ (10^−7^ M) (*n* = 6). mRNA expression determined by real-time PCR, expression relative to *b-2-microglobulin (B2MG)* expression. Protein expression determined by western blot, upper panel: representative immunoblot; lower panel: densitometry of protein expression relative to β-actin expression. Values are expressed as means ± SEM in arbitrary units (AU). Statistical differences with regard to control are marked with asterisks (*p < 0.05; **p < 0.01; ***p < 0.001); Statistical differences with regard to PGF_2α_ are marked with dollars (^$^*p* < 0.05).

In the last experiment we tested the extent of which TSP1 and CD36 are modulated by Nodal. We confirmed that mRNA and protein levels of TSP1 and CD36 were increased after Nodal treatment (Figure 5A, *p* < 0.05, Figure 5B, *p* < 0.01; Figures 5C,D, *p* < 0.05). Also, PGF_2α_ treatment upregulated mRNA and protein of TSP1 (Figure 5A, *p* < 0.001, Figure 5B, *p* < 0.01), and exclusively augmented CD36 protein (Figure 5D, *p* < 0.05). Furthermore, we tested the effect of loss of Nodal and TGFβ1 activity in PGF_2α_ action. When we blocked Nodal and TGFβ1 signaling pathway with SB, PGF_2α_ upregulation of TSP1 and CD36 was abolished (Figures 5E,F, *p* < 0.05).

## Discussion

In the present report we investigate the putative involvement of Nodal in angioregression during luteolysis in the mare. Facing the inexistence of a reliable *in vitro* system with equine luteal endothelial cells, we decided to use mid CL explants in order to instigate the extent of which Nodal luteolytic role involves the activation of vasoactive mediators. Indeed, our previous studies on luteal steroidogenic cells revealed that a great number of the endothelial cells are lost during cell isolation (15, 16). Overall, with mid CL explants we lose cell specificity on the response to our treatments, but we benefit from the contribution of the microvasculature to the response obtained. Thus, we studied the interaction between Nodal and HIF1α and TSP1/CD36 in mid CL explants, two vasoactive systems previously shown to play a role in CL luteolysis (9, 10).

We started the study assessing the effect of Nodal treatment on the endothelial cell marker CD31 and, vice-versa, testing the action of conventional proangiogenic factors like VEGFA and FGF on Nodal signaling regulation in mid CL explants. Members of TGFβ family can play either proangiogenic or antiangiogenic roles (20, 21). For instance, Geng and co-workers evidenced that TGFβ1 decreased VEGFA expression via Smad3P activation in colon cancer cells (22). Presently, we found that Nodal played an inhibitory role on CD31 protein, revealing for the first time the antiangiogenic properties of Nodal in the CL. Indeed, the interaction between TGFβ family and CD31 has been previously reported, particularly under the regulation of endothelial-to-mesenchymal transformation (23). Similar events appear to take place in the CL undergoing luteolysis, as the consistent proliferation of luteal fibroblast was shown to be mediated by TGFβ1 in bovine CL (24). Importantly, our recent studies also revealed the effect of TGFβ1 on equine functional and structural luteolysis (3, 25), reiterating the importance of the TGFβ ligands Nodal and TGFβ1 for equine CL regression. Next, we treated the mid CL explants with the proangiogenic factors VEGFA and FGF, and found that only VEGFA treatment showed a trend on Alk7 downregulation. Interestingly, Alk7 can be seen as an important intracellular mediator of Nodal signaling, once its protein expression profile mimics the one of Nodal throughout the luteal phase [(2); Figure 2E]. Overall, these results suggested the involvement of Nodal in angioregression and encouraged us to further instigate alleged antiangiogenic properties of Nodal during luteolysis in the mare.

Vascular regression is one of the main features of luteolysis and was primarily linked to PGF_2α_ activity (26). Consequently, O_2_ supply in the CL is decreased both physiological luteolysis or after *in vivo* PGF_2α_ treatment (8, 27, 28). Indeed, it was shown that low O_2_ concentration promoted functional luteolysis through inhibition of P4 synthesis, and induced apoptosis and structural regression (8, 9). Therefore, we interrogated if Nodal mediates HIF1α activity, and conversely if O_2_ tension in the CL is able to modulate Nodal signaling expression. First, we characterized the expression of HIF1α in fresh cyclic CL. Despite increased *HIF1*α mRNA levels in early, and mid CL, no changes were found in HIF1α protein. Nonetheless, HIF1α was clearly expressed in mid CL, and a similar expression pattern was found for both Nodal and Alk7. This suggested the availability of these three proteins in mid CL, the time of luteolysis initiation (2, 6, 29). Furthermore, other studies reported Nodal responsiveness to hypoxia, like in melanoma cancer cells (30) and breast cancer cells (31). Also, in glioma cells Nodal was shown to increase HIF1α activity (32). Accordingly, the incubation of our explants in hypoxia (5% O_2_) significantly increased Nodal protein, as well as Smad3P levels. Additionally, after the *in vitro* treatment of mid CL explants with Nodal we verified the amplification of HIF1α protein. Taken together, these results suggested that Nodal not only activated HIF1α, but also was amplified in equine mid CL under low O_2_ tension, which itself represents a feature of luteolysis (8, 27, 28).

A major target of HIF1α is the VEGFA (33), a proangiogenic protein which expression is downregulated in equine regressing CL (34). We challenged our hypothesis, and analyzed the expression of VEGFA in mid CL explants treated with Nodal. Indeed, we found a dose-dependent response to Nodal treatment, in which the lowest dose of Nodal (0.1 ng/mL) increased *VEGFA* mRNA and Nodal 0.1 and 1 ng/mL showed a tendency to upregulate VEGFA protein (*p* = 0.06). However, no effect was seen for Nodal 10 ng/mL, the luteolytic dose, on both mRNA and protein of VEGFA. Despite out of the scope of the present study, one should not exclude a putative proangiogenic action of Nodal in early CL. As mentioned above, TGFβ family members can play either angiogenic or anti-angiogenic roles (21, 22, 24), regarding the physiological context. In fact, Nodal was shown to promote vascularization in breast cancer cells (31), despite its role in CL establishment being unknown. Importantly, it remains clear that the activation of HIF1α by Nodal was done exclusively at 10 ng/mL (Supplementary Figure 2), the treatment dose previously shown to induce functional luteolysis (2). Also, Nodal protein expression profile in cycling CL denotes a sharp rise in mid CL, which supports the idea of higher levels of Nodal being required for mediation of its luteolytic actions. Thus, one may conclude the crosstalk between Nodal and HIF1α in mid CL during luteolysis activation does not imply VEGFA activity.

In the last experiment we explored the interaction between Nodal and another anti-angiogenic system, the TSP1/CD36 pathway (11). Importantly, TSP1 has been shown to be a downstream factor upregulated by HIF1α during hypoxia (35). Furthermore, the luteolytic role of TSP1 has been well-documented before (10). Indeed, we have previously demonstrated that tumor necrosis factor-α anti-angiogenic role comprised TSP1 and its receptor CD36 activation in equine luteal cells (16). Furthermore, in bovine CL, PGF_2α_ induced-luteolysis mediated the upregulation of TSP1 and CD36 (10, 13, 36, 37), a finding which is in agreement with our former results. In the present study, we reported the upregulation of TSP1 and CD36 by Nodal in *in vitro* explants of equine mid CL. These findings further uncovered the intricacies of molecular regulation of luteolysis. We can now consider the interactions in mid CL between O_2_ levels, HIF1α activity, and Nodal signaling as a relevant step for luteolysis activation, which in turn supports the TSP1/CD36 anti-angiogenic activity.

Our last observation made evident the importance of Nodal and TGFβ1 signaling components on PGF_2α_ upregulation of TSP1 and CD36 proteins. In the present study, we used both PGF_2α_ and LH treatments as a positive controls for our culture system. Expectedly, PGF_2α_ amplified both TSP1 mRNA and protein and CD36 protein, as seen before in bovine CL (10, 13, 36, 37). However, no changes were seen on *CD36* mRNA. The absence of agreement between CD36 mRNA and protein level can be eventually linked to mRNA half-life, which can be shorter than 24 h, or post-translational processing of the RNA (38, 39). Conversely, when we pharmacologically blocked Nodal and TGFβ1 receptor Alk4, Alk5, and Alk7 with SB, the effect of PGF_2α_ was abolished. Our previous studies on functional luteolysis and P4 inhibition have made clear the supportive role of Nodal on PGF_2α_-induced functional luteolysis (2). Presently, we confirmed also that PGF_2α_ luteolytic amplification of TSP1/CD36 requires Nodal and TGFβ1 active signaling. This definitely highlights the prominent role of Nodal in the luteolytic cascade.

During luteolysis, PGF_2α_ orchestrates the interactions between TSP1 and TGFβ1 (10). Our previous results demonstrated the importance of the crosstalk between PGF_2α_ and Nodal/ TGFβ1 (2, 3). Furthermore, the link between the main luteolysin and HIF1α was also shown to mediate functional and structural regression of the CL (8). Thus, our present findings invite us to propose a luteolytic network, in which under the regulatory action of PGF_2α_, Nodal acts on two important anti-angiogenic systems, the HIF1α and TSP1/CD36, to support vascular regression during CL regression. Furthermore, taking into consideration the previously documented involvement in functional luteolysis (2), we may now consider too the involvement of Nodal in the modulation of anti-angiogenic factors during luteolysis in mares. To conclude, we made an evidence for the possible interaction between Nodal and HIF1α, as well as Nodal signaling sensitivity to hypoxia in the CL. Additionally, Nodal not only upregulated TSP1/CD36 system, but was also shown to be required for PGF_2α_-induced upregulation of TSP1 and CD36 in equine CL. These results suggest the involvement of Nodal in angioregression during luteolysis in the mare and deserve being further studied.

## Data Availability Statement

The datasets generated for this study are available on request to the corresponding author.

## Ethics Statement

The animal study was reviewed and approved by Local Animal Care and Use Committee in Olsztyn, Poland (Agreement No. 51/2011).

## Author Contributions

EW and KW: conception and design, acquisition of data, analysis and interpretation of data, and drafting and revising the article. DS and GF-D: analysis and interpretation of data and drafting or revising the article. AG: conception and design, analysis and interpretation of data, and drafting and revising the article.

### Conflict of Interest

The authors declare that the research was conducted in the absence of any commercial or financial relationships that could be construed as a potential conflict of interest.
