# Supplementary material for: The Interaction Between Nodal, Hypoxia-Inducible Factor 1 Alpha, and Thrombospondin 1 Promotes Luteolysis in Equine Corpus Luteum
Source: Front Endocrinol (Lausanne). 2019 Oct 1;10:667. doi: 10.3389/fendo.2019.00667 (PMC6779822; doi:10.3389/fendo.2019.00667)
Supplement: Supplementary file 1 [file Table_1.DOCX]

Fig. suppl. 1 Walewska, Wołodko et al. 2019


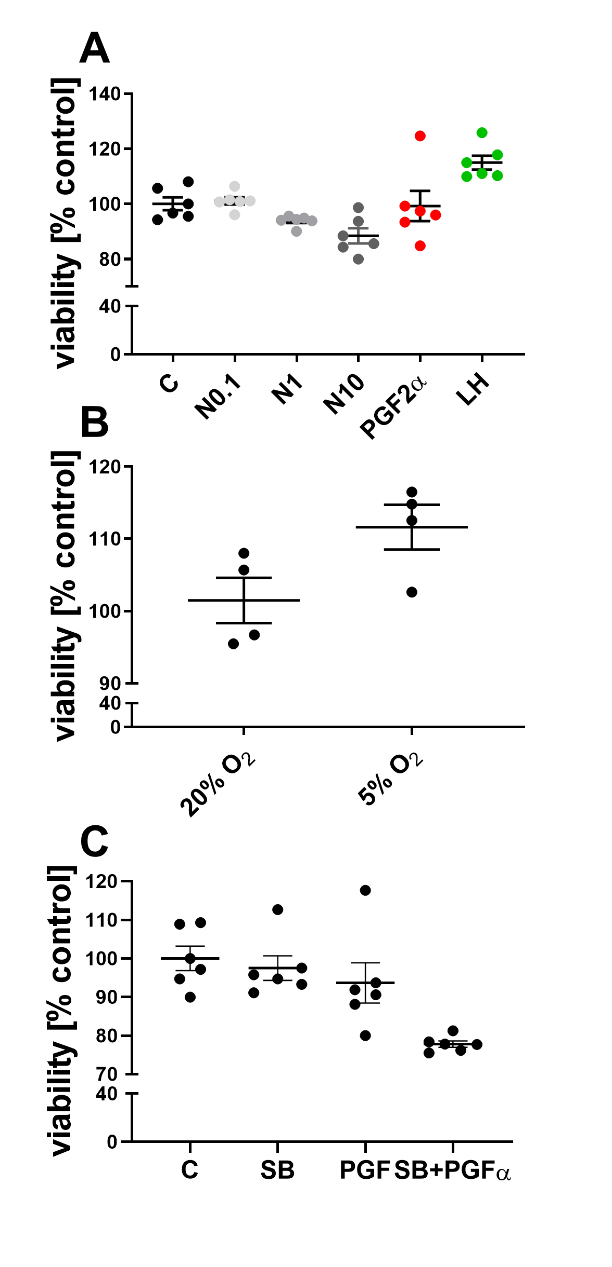


**Fig. suppl. 1** **Equine luteal explants viability assay** (A) Viability of mid CL explants after *in vitro* stimulation with Nodal (concentrations from 0.1 to 10 ng/mL), prostaglandin F2α (PGF2α, 10^-7^ M); and luteinizing hormone (LH, 10 ng/mL). (B) Explants viability exposed to 20% and 5% of oxygen. (C) Viability of mid CL explants after *in vitro* stimulation with SB (10 μM), PGF2α (10^-7^ M) and PGF2α (10^-7^ M) with SB (10 μM). Results expressed as % of control. (n=6 for A and C; n=4 for B). Values are expressed as means ± SEM.


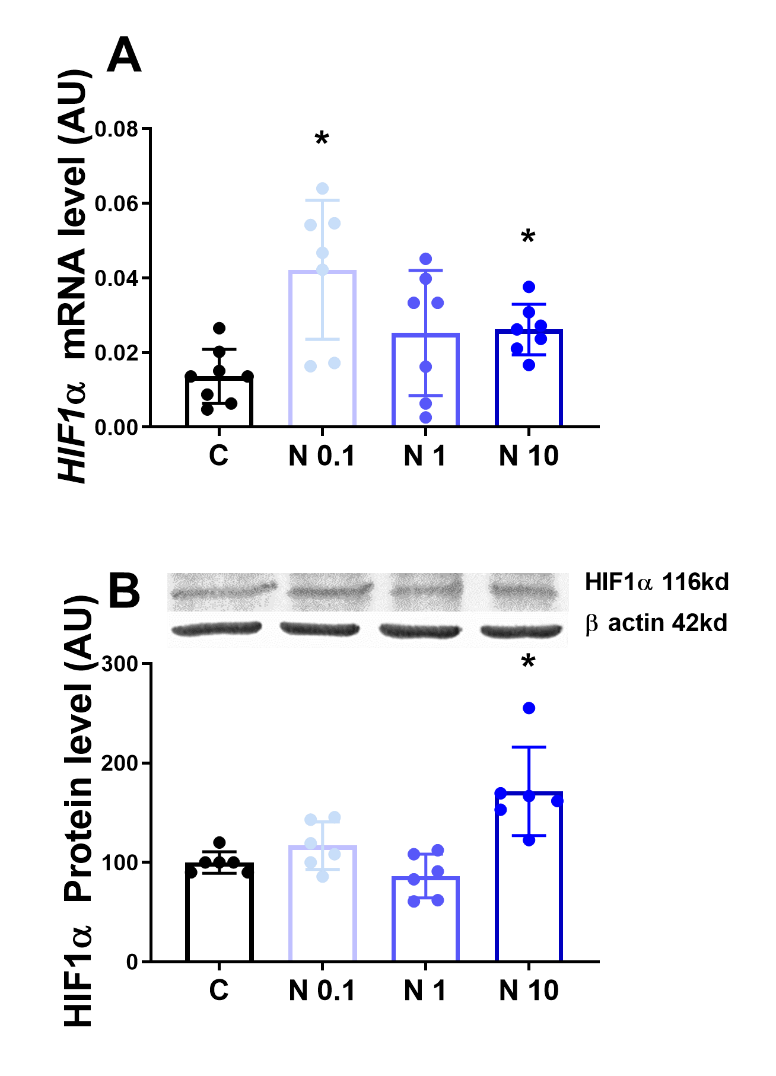
Fig. suppl. 2 Walewska, Wołodko et al. 2019

**Fig. suppl. 2 HIF1α mRNA and protein expression after *in vitro* culture of mid CL explants** Expression of (A) *hypoxia inducible factor 1* *α (HIF1α)* mRNA, (B) HIF1α protein after 24h culture: (i) no factor (negative control); (ii) Nodal (0,1 ng/mL); (iii) Nodal (1 ng/mL); (iv) Nodal (10 ng/mL) (n=6). mRNA expression determined by real-time PCR, expression relative to *β−2-microglobulin (B2MG)* expression. Protein expression determined by western blot, upper panel: representative immunoblot; lower panel: densitometry of protein expression relative to β-actin expression. Values are expressed as means ± SEM in arbitrary units (AU). Statistical differences are marked with asterisks (* p<0.05).
